# The complete mitochondrial genome of *Harpago chiragra* and *Lambis lambis* (Gastropoda: Stromboidea): implications on the Littorinimorpha phylogeny

**DOI:** 10.1038/s41598-019-54141-x

**Published:** 2019-11-27

**Authors:** Dianhang Jiang, Xiaodong Zheng, Xiaoqi Zeng, Lingfeng Kong, Qi Li

**Affiliations:** 1 Institute of Evolution & Marine Biodiversity (IEMB), Ocean University of China, Qingdao, 266003 China; 2 Key Laboratory of Mariculture, Ministry of Education, Ocean University of China, Qingdao, 266003 China

**Keywords:** Molecular evolution, Evolutionary biology

## Abstract

The complete mitochondrial genomes of *Harpago chiragra* and *Lambis lambis* (Strombidae) were determined with the size of 15,460 bp and 15,481 bp, respectively, and both sequences contained 13 protein-coding genes, 22 tRNAs, and two rRNAs. *H. chiragra* and *L. lambis* have similar mitochondrial features, corresponding to typical gastropod mitochondrial genomes, such as the conserved gene order, a high A + T content (66.22% for *H. chiragra* and 66.10% for *L. lambis*), and preference for A + T-rich codons. The start or termination codon of same protein-coding gene in *H. chiragra* was consistent with that in *L. lambis*, except for the termination codon of *cox1* gene (TAG for *H. chiragra* and TAA for *L. lambis*) and the start codon of *nad4* (GTG for *H. chiragra* and ATG for *L. lambis*). Pairwise sequence alignments detected different degrees of variations in *H. chiragra* and *L. lambis* mitochondrial genomes; and the two species had lower levels of genetic distance (0.202 for nucleotide sequence) and closest relationships as compared to *Strombus gigas* and *Oncomelania hupensis*. The 13 partitioned nucleotide sequences of protein coding genes of *H. chiragra* and *L. lambis* were aligned with representatives of the main lineages of gastropods and their phylogenetic relationships were inferred. *H. chiragra* and *L. lambis* share the same gene order as Littorinimorpha species, except Vermetoidea, which demonstrate a gene rearrangement in species. The reconstructed phylogeny supports three major clades within Littorinimorpha: 1) Stromboidea, Tonnoidea, Littorinoidea, and Naticoidea, 2) Rissooidea and Truncatelloidea, and 3) Vermetoidea. In addition, a relaxed molecular clock calibrated with fossils dated the diversification of Strombidae near 112 (44–206) Mya and a possible radiation is detected to occur between 45–75 Mya, providing implications to understand the Cenozoic replacement event (65–135 Mya) of Aporrhaidae by Strombidae.

## Introduction

Molecular phylogenetic analyses provided a different approach compared with traditional morphological methods to estimate the relationships among species based on the topological hypotheses^1–3^. The mitochondrial DNA has a high rate of base substitution and lacks of recombination during inheritance; besides it possesses an unique transmission mode named doubly uniparental inheritance (DUI) in molluscs^4,5^. Hence mitochondrial genomic analyses was proved as a valid molecular tool in constructing phylogenies, and has been used for phylogenetic analyses in various taxa^6,7^.

The derived phylogenetic relationships based on molecular data may disagree with the evolutionary hypothesis proposed using morphological data^2,3^. Neogastropoda was widely accepted as a monophyletic group based on morphological characters; however, Tonnoidea was placed into Neogastropoda based on the molecular analyses^8–11^, which contradicted the monophyletic status of Neogastropoda. Among Littorinimorpha, Vermetidae is a peculiar snail family that shows a high rate of gene rearrangement^12^. Based on the molecular phylogenetic analyses, Vermetidae were regarded as the sister group of the other species in Caenogastropoda,; however, this is opposite to the morphological evidence^13^. Hence, the relative position of Vermetidae in the mitochondrial phylogenetic analyses has been considered spurious, although the relationship was highly supported in previous molecular phylogenetic analyses^14,15^.

Strombidae species are important molluscs in shallow water of tropical and subtropical areas from past time until now^16–19^. Species in Stromboidae varied greatly in shell shapes, which results in high morphological diversity^19,20^. *Strombus* (Linné,1758) and *Lambis* (Röding, 1798) are the two most abundant genera in Strombidae and were once regarded as the only two genera in strombids^21^. However, based on the fossil record and molecular phylogenetic analyses, the genus *Strombus* is justified to be subdivided into several separate genera^20,21^.

Based on the paleontological studies, Strombidae probably originated from Aporrhaidae during Cenomanian-Turonian, and evolved at low diversities during the rest of the Cretaceous^19^. During the course of evolution, whereas Aporrhaidae species underwent K/T mass extinction in late Cretaceous, a major genera and species radiation in Strombidae occurred during the early Cenozoic and continued to the Pliocene^19,22,23^.

In the present study, we determined the complete mitochondrial genomes of *H. chiragra* and *L. lambis*, and analyzed the genomic features of the two species, including their structural characters and nucleotide composition. In early taxonomical studies, *H. chiragra* shared close relationships with *L. lambis* in Strombidae, yet this was mainly based on their similar tissue anatomies (e.g. egg masses, and radulae)^18^, regardless of the great morphological difference in adults. To valid the taxonomy relation between *H. chiragra* and *L. lambis*, we attempt to determine their phylogenetic relationships based on the mitochondrial genomes. Thus, a robust phylogeny based on the concatenated 13 protein coding genes of 15 Littorinimorpha species was constructed. These data provides a framework for further evolutionary studies among Littorinimorpha.

## Materials and Methods

### Specimen and mitochondrial DNA extraction

Individuals of *H. chiragra* and *L. lambis* were collected from the coastal waters of Quanfu Island, South China Sea. Total genomic DNA was extracted from the foot muscle using a modified standard phenol-chloroform procedure^24^ and then stored at −20 °C.

### Determination of partial sequences

Short fragments of *cox1* gene were PCR amplified using the universal primers LCO-1490/HCO-2198^25^. Based on the reference genome of *S. gigas*^26^, primers were designed using Primer Premier 5^27^ to amplify short fragments of *atp6*, *cox3*, and *cytb* (Supplementary Table S1). Long PCR primers were designed to amplify the regions between the genes based on the partial sequences obtained.

### PCR amplification and sequencing

PCR was performed in a 30 μL reaction mixture containing 3 μL of dNTPs (2.5 mM each), 3 μL of 10 × LA buffer (Mg^2+^), 1 μL of template DNA (100 ng/μL), 1 μL of each forward and reverse primer, and 0.5 μL of TaKaRa LA-*Taq* DNA polymerase. The thermal cycling conditions are: 94 °C for 3 min followed by 35 cycles of denaturing at 94 °C for 30 s, annealing at 62 °C for 30 s, and extension at 68 °C for 5 min, with a final extension step of 72 °C for 10 min. PCR products were purified using an EZ-10 Spin Column DNA Gel Extraction Kit (Sangon Biotech), and then directly sequenced using the primer walking method. DNA sequencing was performed on an ABI PRISM 3730 (Applied Biosystems) automatic sequencer.

### Genome annotation and sequence analysis

Sequence assembly was performed using the Seqman program, DNASTAR (http//www.DNASTAR.com). The annotations of protein-coding genes were conducted using ORF Finder (https://www.ncbi.nlm.nih.gov/orffinder/) with invertebrate mitochondrial genetic code. The transfer RNA (tRNA) genes were identified using ARWEN (http://130.235.46.10/ARWEN/) and MITOS web servers (http://mitos.bioinf.uni-leipzig.de/index.py) using the mitochloroplast or invertebrate gene code and default search code.

The gene annotation of *rrnL* and *rrnS* were conducted using BLAST searches (https://blast.ncbi.nlm.nih.gov/Blast.cgi) by identifying their similarity to gene sequences of *S. gigas* and *Conomurex luhuanus*. The A + T content values were computed using MEGA 6.06^28^ and GC and AT skews were calculated according to the formulae described before^29^, AT skew = (A − T)/(A + T); GC skew = (G − C)/(G + C), where A, T, G, and C are the occurrences of the four nucleotides. The relative synonymous codon usage (RSCU) values of each protein coding gene were calculated using MEGA 6.06^28^. The number of base substitutions per site between *H. chiragra L. lambis*, *S. gigas*, and *C. luhuanus* were calculated in MEGA 6.06^28^ using the Kimura 2-parameter model^30^.

### Phylogenetic analyses

The phylogenetic analyses were based on the concatenated nucleotide and amino acid alignments of thirteen protein-coding genes in seventeen complete mitochondrial genomes, including *H. chiragra*, *L. lambis* and 13 other available mitochondrial genomes of Littorinimorpha (Supplementary Table S2). Besides, *Tegula lividomaculata* and *Tegula brunnea* from the order Trochida served as outgroup. The thirteen-partitioned nucleotide and amino acid sequences of the protein-coding genes were aligned using MAFFT^31^ with automatic selection of alignment algorithm. Then the alignments were treated with Gblocks^32,33^ using default parameters, and the ambiguously aligned regions were removed from the analyses. Multiple gene alignments were concatenated using PhyloSuite^34^. Then we evaluated the saturation in the codon-based data sets of thirteen protein coding genes in DAMBE7^35^, and the results showed that the DNA sequences were unsaturated in 1^st^-2^nd^-3^rd^ and 3^rd^ codon sites. The best-fit partition schemes of amino acid and nucleotide sequences were selected using PatitionFinder 2.1.1^36^. Two methods were used to perform the phylogenetic analyses: Maximum Likelihood (ML) and Bayesian inference (BI). ML analysis was conducted using RAxML^37^ web server on the CIPRES Science Gateway V.3.3 (http://www.phylo.org/index.php/) based on the partitioned nucleotide alignments, with GTR + G substitution model and 1,000 bootstraps for node reliability estimation. Bayesian analyses were conducted in MrBayes^38^ for 200 million generations (sampling every 1000 generations) based on the partitioned nucleotide and amino acid alignments. All parameters were checked using Tracer v1.5^39^. The first 50,000 trees were discarded as burnin, and the remaining sampled trees were used to estimate the Bayesian posterior probabilities.

### Estimate of divergence time

The estimation of divergence time of the major Littorinimorpha lineages were conducted using BEAST v.1.7.5^39^ based on the partitioned amino acid sequences of 13 protein coding genes. A lognormal relaxed-clock model was selected as the molecular clock model. A Yule process of speciation was chosen for the tree prior. The final Markov chain was set to 100 million generations, sampling every 10,000 generations. The effective sample size of all parameters was above 200. The convergence of the chains was checked with Tracer v.1.5^39^, and the first 1,000 generations sampled were discarded as part of the burn-in process.

The posterior distribution of the estimated divergence times was specified based on the prior fossil knowledge. Two calibration points were selected, using a normal distribution of prior probability: 342.8 Mya was used as prior divergence time for Vermetoidea based on the Paleocene fossil collection in Belgium and the United Kingdom^40^, and the prior divergence time of Truncatellidae was set as 66.04 Mya according to the oldest fossil record of Paleocene in Belgium^41–43^. Besides, the divergence time of *Tegula* was 85 Mya based on the Cretaceous fossil record in United States^41^, and this point was used to cross-validate the accuracy of the dated tree.

## Results and Discussion

### Genome organization of *H. chiragra* and *L. lambis*

**Figure 1:**
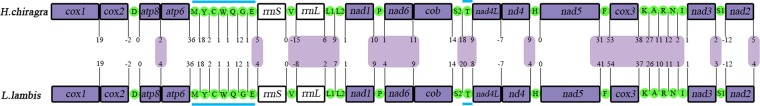
Linear comparison of the gene organization of *H. chiragra* and *L. lambis* mitochondrial genomes. The blue lines indicated genes coded by the minor strand. Positive numbers mean the length in bp of non-coding regions between genes and negative numbers represent overlapping nucleotides between genes.

The complete mitochondrial genome sequences of *H. chiragra* and *L. lambis* are 15,460 bp and 15,481 bp, respectively (Tables 1, 2), and both contain 13 protein coding genes (PCGs), 22 tRNAs and two rRNAs (Fig. 1). This (+) strand encodes for *trnD*, *trnV*, *trnL*, *trnL*, *trnP*, *trnS*, *trnH*, *trnF* and the cluster KARNI (*trnK*, *trnA*, *trnR*, *trnN*, and *trnI*) and *trnS*. The (−) strand encodes for the cluster MYCWQGE (*trnM*, *trnY*, *trnC*, *trnW*, *trnQ*, *trnG*, and *trnE*) and *trnT* (Fig. 1). Four overlaps between adjacent genes were detected in *H. chiragra* and *L. lambis*, in addition, another region between *atp8* and *trnV* was found only in *H. chiragra*, but not in *L. lambis* (Fig. 1). The lengths of genes (including PCGs, tRNAs and rRNAs) and intergenic nucleotides are 15129 bp, 331 bp for *H. chiragra* and 15146 bp, 335 bp for *L. lambis*, respectively (Tables 1, 2), in which the gene length of the overlapping nucleotides was counted once.

**Table 1:** Organization of the mitochondrial genome of *Lambis lambis* (15,481 bp).

Gene	From	To	Size (nts)	Size (aa)	Intergenic nucleotides	Start codon	Termination codon
*cox1*	1	1536	1536	511	4	ATG	TAA
*cox2*	1556	2242	687	228	19	ATG	TAA
*trnD*(gac)	2241	2308	68		−2		
*atp8*	2309	2467	159	52	0	ATG	TAA
*atp6*	2472	3167	696	232	4	ATG	TAA
*trnM*(atg)	3204	3271	68		36		
*trnY*(tac)	3290	3355	66		18		
*trnC*(tgc)	3358	3422	65		2		
*trnW*(tga)	3424	3490	67		1		
*trnQ*(caa)	3492	3553	62		1		
*trnG*(gga)	3566	3632	67		12		
*trnE*(gaa)	3634	3703	70		1		
*rrnS*	3708	4693	986		4		
*trnV*(gta)	4694	4760	67		0		
*rrnL*	4753	6147	1395		−8		
*trnL1*(cta)	6150	6218	69		2		
*trnL2*(tta)	6226	6294	69		7		
*nd1*	6296	7237	942	313	1	ATG	TAG
*trnP*(cca)	7247	7315	69		9		
*nad6*	7320	7826	507	168	4	ATG	TAA
*cytb*	7836	8975	1140	379	9	ATG	TAA
*trnS2*(tca)	8990	9055	66		14		
*trnT*(aca)	9076	9141	66		20		
*nad4l*	9150	9446	297	98	8	ATG	TAG
*nad4*	9440	10813	1374	457	−7	ATG	TAA
*trnH*(cac)	10818	10884	67		4		
*nad5*	10885	12612	1728	575	0	ATG	TAA
*trnF*(ttc)	12654	12725	72		41		
*co3*	12780	13559	780	259	54	ATG	TAA
*trnK*(aaa)	13597	13666	70		37		
*trnA*(gca)	13693	13763	71		26		
*trnR*(cga)	13774	13842	69		10		
*trnN*(aac)	13854	13923	70		11		
*trnI*(atc)	13925	13992	68		1		
*nad3*	13994	14347	354	117	1	ATG	TAA
*trnS1*(agc)	14351	14418	68		3		
*nad2*	14407	15477	1071	356	−12	ATC	TAG

**Table 2:** Organization of the mitochondrial genome of *Harpago chiragra* (15,460 bp).

Gene	From	To	Size (nts)	Size (aa)	Intergenic nucleotides	Start codon	Termination codon
*cox1*	1	1536	1536	511	5	ATG	TAG
*cox2*	1556	2242	687	228	19	ATG	TAA
*trnD*(gac)	2241	2308	68		−2		
*atp8*	2309	2467	159	52	0	ATG	TAA
*atp6*	2470	3165	696	231	2	ATG	TAA
*trnM*(atg)	3202	3269	68		36		
*trnY*(tac)	3288	3353	66		18		
*trnC*(tgc)	3356	3420	65		2		
*trnW*(tga)	3422	3488	67		1		
*trnQ*(caa)	3490	3551	62		1		
*trnG*(gga)	3564	3630	67		12		
*trnE*(gaa)	3632	3701	70		1		
*rrnS*	3707	4684	978		5		
*trnV*(gta)	4685	4751	67		0		
*rrnL*	4737	6127	1391		−15		
*trnL1*(cta)	6134	6202	69		6		
*trnL2*(tta)	6212	6280	69		9		
*nd1*	6282	7223	942	313	1	ATG	TAG
*trnP*(cca)	7234	7301	68		10		
*nad6*	7303	7809	507	168	1	ATG	TAA
*cytb*	7821	8960	1140	379	11	ATG	TAA
*trnS2*(tca)	8975	9040	66		14		
*trnT*(aca)	9059	9125	67		18		
*nad4l*	9135	9431	297	98	9	ATG	TAG
*nad4*	9425	10798	1374	457	−7	GTG	TAA
*trnH*(cac)	10808	10874	67		9		
*nad5*	10875	12602	1728	575	0	ATG	TAA
*trnF*(ttc)	12634	12702	69		31		
*co3*	12756	13535	780	259	53	ATG	TAA
*trnK*(aaa)	13574	13643	70		38		
*trnA*(gca)	13671	13742	72		27		
*trnR*(cga)	13754	13822	69		11		
*trnN*(aac)	13835	13901	67		12		
*trnI*(atc)	13904	13971	68		2		
*nad3*	13973	14326	354	117	1	ATG	TAA
*trnS1*(agc)	14329	14396	68		2		
*nad2*	14385	15455	1071	356	−12	ATC	TAG

**Figure 2:**
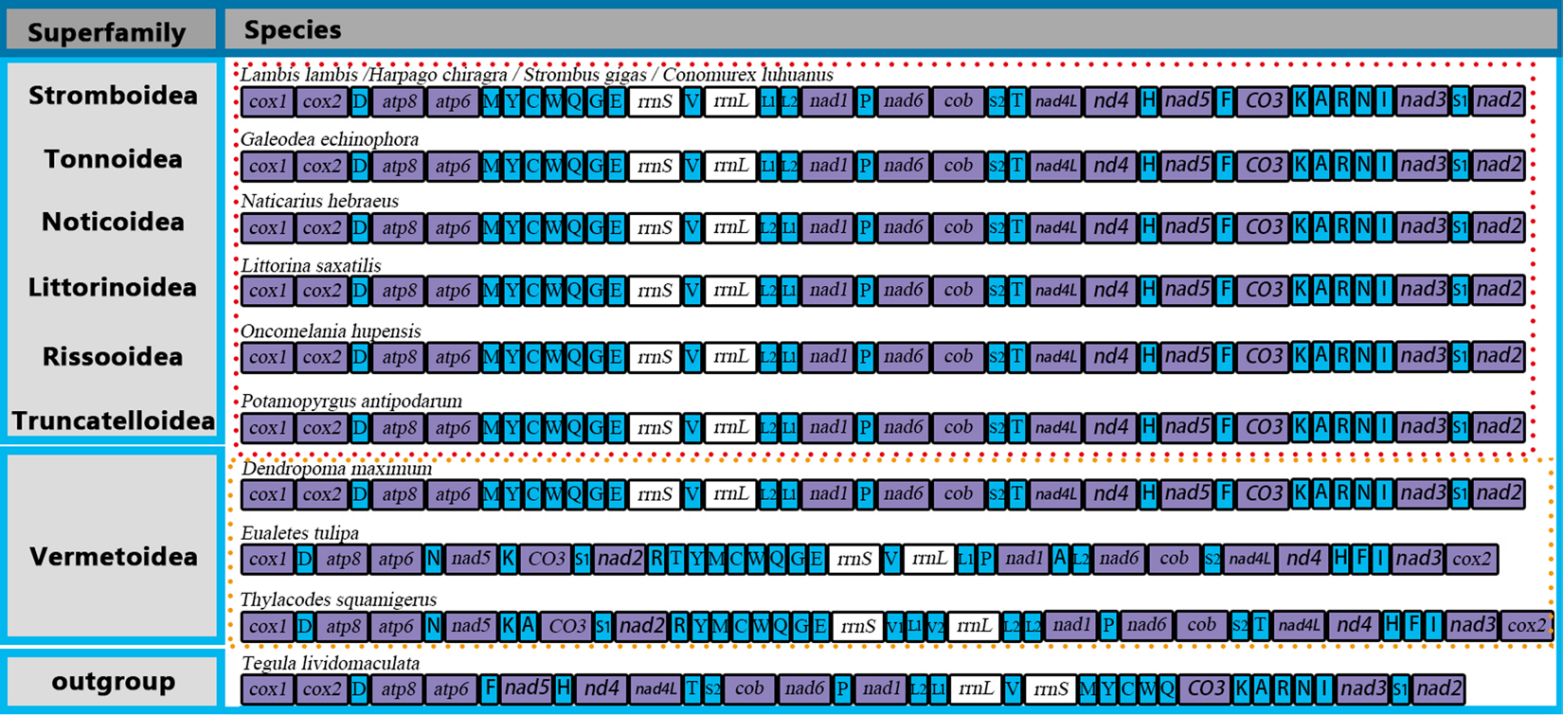
Linear comparisons of the organization of the mitochondrial genomes of Littorinimorpha.

The organization of the *H. chiragra* and *L. lambis* mitochondrial genomes was compared with that of other sepcies in Littorinimorpha (Fig. 2). Among the gastropod species, mitochondrial genomes are estimated to show high rates of gene rearrangement between major lineages^44^. However, the gene orders of the two newly sequenced mitochondrial genomes were similar to the consensus gene order shared by most previously published species from Littorinimorpha^26,45^(Fig. 2).

### Nucleotide composition

The overall base compositions of the mitochondrial genomes on the (+) strand were both biased toward A and T. For *H. chiragra*, the nucleotide content was found to be A = 28.26%, T = 37.6%, C = 16.57%, and G = 17.21%. For *L. lambis*, the nucleotide content was A = 28.61%, T = 37.49%, C = 16.50%, and G = 17.40% (Table 3). For the entire mitochondrial genomes, the AT and GC-skews on the (+) strand were −0.128 and 0.019 for *H. chiragra* and −0.134 and 0.026 for *L. lambis*, respectively (Table 3).

**Table 3:** AT-content, AT-skew, and GC-skew for mitochondrial genes of *H. chiragra* and *L. lambis*.

Feature	(A + T) %		AT skew		GC skew	
	*H. chiragra*	*L. lambis*	*H. chiragra*	*L. lambis*	*H. chiragra*	*L. lambis*
Whole genome	66.22	66.10	−0.13	−0.13	0.02	0.03
Protein coding genes	65.68	65.68	−0.19	−0.20	−0.01	0.01
*atp6*	65.80	67.10	−0.26	−0.31	−0.15	−0.11
*atp8*	71.60	67.92	−0.17	−0.19	0.04	−0.02
*cox 1*	62.63	63.09	−0.20	−0.23	0.05	0.07
*cox 2*	63.46	65.00	−0.09	−0.14	0.05	0.09
*cox 3*	60.38	60.90	−0.24	−0.23	0.14	0.15
*cytb*	65.00	63.77	−0.21	−0.23	−0.04	−0.04
*nad1*	68.68	66.67	−0.23	−0.22	0.03	0.04
*nad2*	68.53	68.35	−0.18	−0.21	0.15	0.12
*nad3*	66.67	63.84	−0.19	−0.20	0.14	0.16
*nad4*	66.67	66.74	−0.16	−0.16	−0.09	−0.09
*nad4L*	69.02	67.00	−0.15	−0.10	0.07	0.04
*nad5*	65.74	66.96	−0.14	−0.14	−0.15	−0.12
*nad6*	68.44	69.23	−0.28	−0.28	−0.15	−0.05
tRNAs	65.88	65.30	0.02	0.03	0.16	0.14
*rrnS*	65.75	65.62	0.06	0.09	0.12	0.09
*rrnL*	68.51	68.24	0.06	0.07	0.14	0.14

The nucleotide composition of the single gene region of *H. chiragra* and *L. lambis* were calculated. The A + T content of protein coding genes (PCGs), tRNA, rRNA, and non-coding regions (NCRs) is similar between *H. chiragra* and *L. lambis* (Table 3). For single genes, similar A + T content was only detected in *cox3* (60%), *nad2* (68%) and *nad4* (66%).

Among the different types of genes, the tRNA genes and rRNA genes of *H. chiragra* and *L. lambis* show positive AT skews, whereas all types of protein coding genes show negative AT skews. Both the tRNA and rRNA genes of *H. chiragra* and *L. lambis* show positive GC skews. Some protein coding genes (*atp6*, *cob*, *nad4*, *nad5*, and *nad6* in *H. chiragra* and *L. lambis* and *atp8* in *L. lambis*) show negative GC skews, while the other types of protein coding genes and RNA genes (tRNA genes and *rrnL*, *rrnS*) show positive GC skews.

### Protein coding genes (PCGs)

Excluding the termination codons, the mitochondrial genomes of *H. chiragra* and *L. lambis* encode 3,744 and 3,745 amino acids, respectively. Comparison between species of start and termination codons of protein coding genes showed that only 2 PCGs initiate or stop with different codons, which were detected in *nad4* gene (initiated with GTG in *H. chiragra* and ATG in *L. lambis*) and *cox*1 gene (stopped with TAG in *H. chiragra* and TAA in *L. lambis*). In addition, 12 PCGs contain the same start (*nad2*: ATC; ATG for the other 11 PCGs) and termination (*nad2*: TAG; *nad1*: TAG; *nad4L*: TAG; TAA for the other 9 PCGs) codons between species (Supplementary Fig. S1).

Pairwise divergence among four Strombidae mitochondrial genomes was calculated based on separate and concatenated protein-coding genes (Supplementary Fig S2). The nucleotide divergence between *H. chiragra* and *L. lambis* was 0.151, which was the lowest genetic divergence measured here, confirming the close relationship between *H. chiragra* and *L. lambis*. *Strombus gigas* and *C. luhuanus* have a nucleotide divergence of 0.351, indicating a relatively distant relationship. Compared with the nucleotide divergence among the four Strombidae mitogenomes, the pairwise divergence values calculated using the amino acid sequences were lower, indicating that synonymous substitutions in protein-coding genes were more frequent than nonsynonymous substitutions.

**Figure 3:**
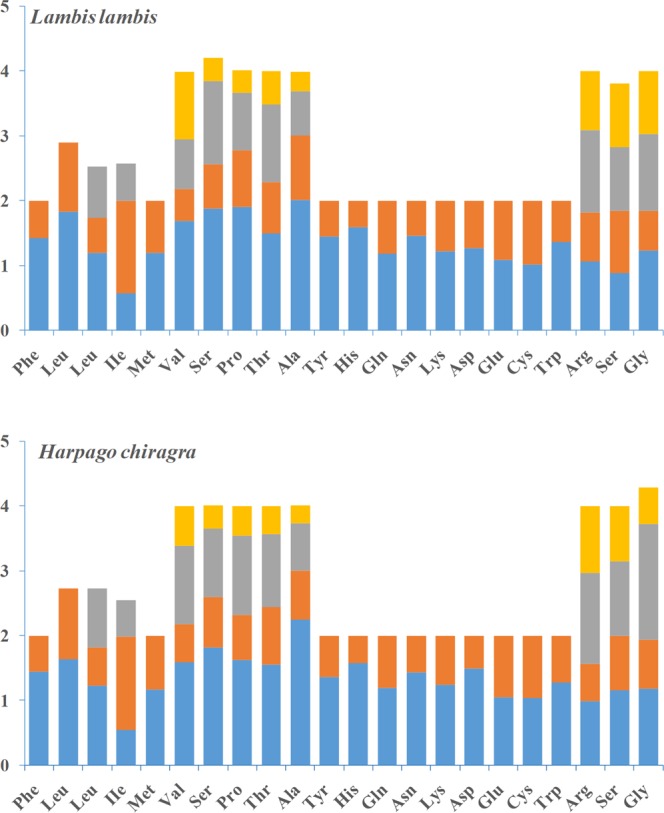
Relative synonymous codon usage (RSCU) of *H. chiragra* and *L. lambis* mitochondrial genomes. The termination codon is not given.

The codon usage of the mitogenomes of *H. chiragra* and *L. lambis* was similar to that of other Strombidae species^26,45^. All codons were used in the mitogenomes of these two species, however the codon frequencies varied between each other. Amino acids encoded by A + T-rich codons are more common than those encoded by G + C-rich codons. The ratio of A + T/G + C-rich codons was 2.61 in *L. lambis*, which was lower than what was found for *H. chiragra* (2.70). The relative synonymous codon usage (RSCU) is different between *H. chiragra* and *L. lambis*, implying their larger genetic difference than previously recognized (Fig. 3). The RSCU also reflected the nucleotide composition bias. For Phe (UUY), the RSCU was 1.42/1.44 for UUU, but only 0.58/0.56 for UUC in the *L. lambis*/ *H. chiragra* mitochondrial genomes, respectively. Amino acids coded by A + T-rich codons are 2.61/2.70 times higher than G + C-rich codons in the *L. lambis*/*H. chiragra*, respectively. The codon usage bias observed in the two snails indicated that the two strands were exposed to different mutational pressures during replication, and it increases the frequency of A + T-rich codons, which is similar to the reports in the vertebrate mitogenomes^46–48^.

### Non-coding regions

There were 34 non-coding regions distributed in the *H. chiragra* and *L. lambis* genomes, 403 bp for *H. chiragra* and 393 bp for *L. lambis* (Fig. 1). The non-coding sequences were generally characterized by short nucleotide fragments, ranging from 1 bp to 53 bp in *H. chiragra* and 1 bp to 54 bp in *L. lambis* among every non-coding fragment. The largest non-coding region was found between the gene *cox3* and *trnF* (53 bp for *H. chiragra* and 54 bp for *L. lambis*). This location was proposed as a candidate to contain the control region in other gastropod mitochondrial genomes^49^. Among the non-coding regions, there were 20 regions with different lengths between *H. chiragra* and *L. lambis* and 14 intervals with same length.

### Phylogenetic analyses

**Figure 4:**
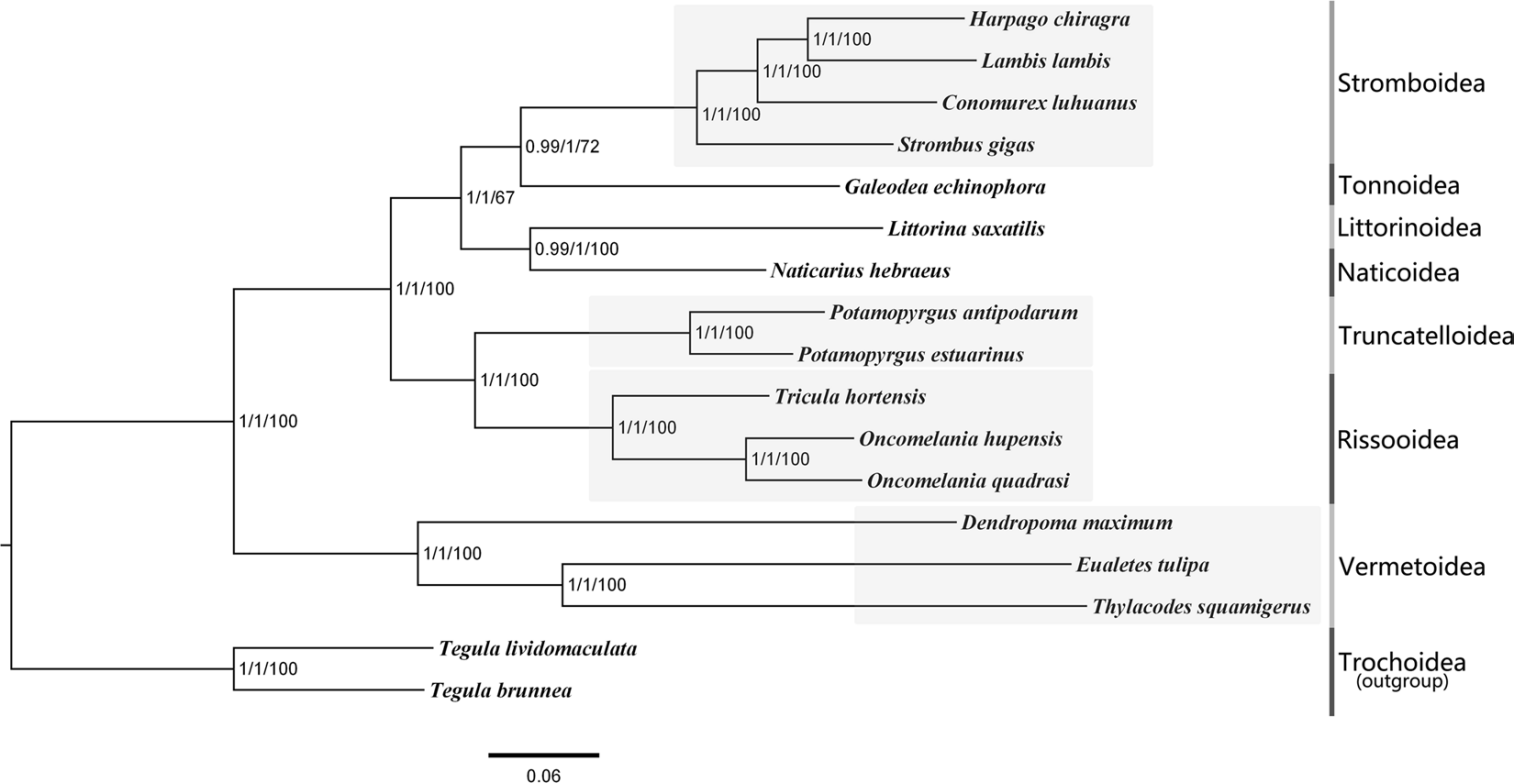
Phylogenetic trees derived from BI and ML analyses based on amino acids and nucleotide sequences of 13 protein coding genes. The first and second numbers at each node are the bootstrap values of posterior probabilities based on aligned nucleotide and amino acids sequences of 13 mitochondrial protein coding genes. The third number is ML bootstrap values based on nucleotide sequences.

The selected partition schemes for phylogenetic analyses were listed in Supplementary Tables S3, S4. The topological structure of the trees inferred by two different methods (ML and BI) was essentially uniform (Fig. 4). All nodes in the BI tree were near 100% supported and the nodes in the ML tree were also highly supported. Within Stromboidea, the phylogenetic tree shows that Strombidae form an independent branch as (*S. gigas* + (*C. luhuanus* + (*L. lambis* and *H. chiragra*))). *L. lambis* is the closest extant relative of *H. chiragra*, and this clade clustered with *S. gigas* and *C. luhuanus*. Research derived from combined phylogenetic analyses of molecular and morphological data has revealed that *Lambis* was monophyletic and *Strombus* was paraphyletic^20^. However, when the cladistics analyses of species in *Lambis* were based solely on morphological characters, the results clustered one *Lambis* species (*L. crocata*) into the outgroups of species^50^, suggesting that *Strombus* is polyphyletic and the *Lambis* is paraphyletic. *Lambis crocata* was not included in the present study since there is no complete mitochondrial genome available for this species. Although lacking a sufficient number of species for a robust phylogenetic analysis, our phylogeny is statistically supported and aims to provide a reasonable framework for further phylogenetic research within Stromboidea.

Within Littorinimorpha, Stromboidea and Tonnoidea clustered together in the same clade, which was then clustered with (Littorinoidea + Naticoidea), as derived by the BI method. Stromboidea, Tonnoidea, Littorinoidea, and Naticoidea form a well-supported clade based on both ML and BI, confirming their close relationship within Littorinimorpha. Rissooidea was sister to Truncatelloidea, which together formed the second major clade. Vermetoidea formed the third independent well-supported clade within Littorinimorpha.

### Estimate of divergence time

**Figure 5:**
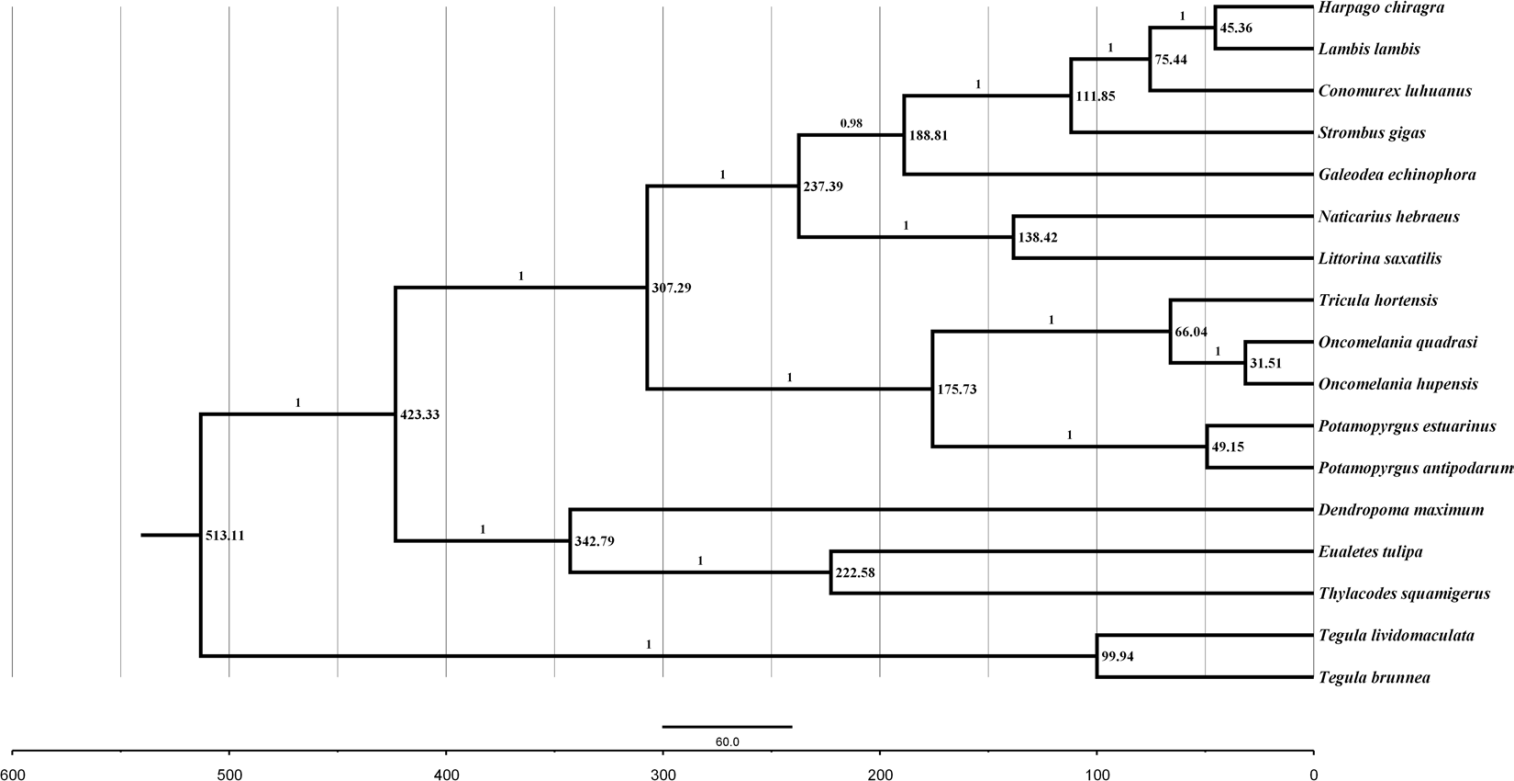
Phylogenetic tree with age estimates of 17 mollusk species based on the mitochondrial dataset and Bayesian relaxed dating methods (BEAST). The numbers next to the nodes are presumably ages. The posterior probability of each node is given above the line. Dates are in millions of years (Mya).

To test the accuracy of the dated tree derived from BEAST, we made a cross-validation using the calibration point of genus *Tegula*. The oldest fossil record of *Tegula* was stated as 85 Mya^41^ and the documented time was coincident with the divergence time of *T. lividomaculata* and *T. brunnea* (13–309 Mya) in present study. Research documented that the number of species increased from five species within the genus *Strombus* during the Eocene (53 Mya) to 40 species till the Late Oligocene and the Miocene (23–36 Mya)^16^, indicating that the species diversification within Strombidae accelerated in the last 36–53 Mya. According to the present dated tree (Fig. 5) the diversification of Strombidae species occurred around 112 (44–206) Mya, and a radiation pattern (accelerated rates of diversification) is detected to occur between 45–75 Mya, which is in agreement with the fossil record in Strombidae. Besides, the diversification pattern of Strombidae species occur between the late Cretaceous and early Paleocene (65–135 Mya), and this might provide implications to understand the Cenozoic replacement event of Aporrhaidae by Strombidae^19,22,23^. Furthermore, to better resolve the phylogenetic relationships and understand the replacement event, more Strombidae and Aporrhaidae mitochondrial genomes should be inserted into the phylogenetic analyses.

## Supplementary information

Dataset 1

## Data Availability

Data is available at Genbank (accession number MH115428, MH122656).
