# Supplementary material for: The complete mitochondrial genome of Harpago chiragra and Lambis lambis (Gastropoda: Stromboidea): implications on the Littorinimorpha phylogeny
Source: Sci Rep. 2019 Nov 27;9:17683. doi: 10.1038/s41598-019-54141-x (PMC6881320; doi:10.1038/s41598-019-54141-x)
Supplement: Supplementary file 1 — Dataset 1 [file 41598_2019_54141_MOESM1_ESM.docx]

The complete mitochondrial genome of *Harpago chiragra* and *Lambis lambis* (Gastropoda: Stromboidea): implications on the Littorinimorpha phylogeny

**Dianhang Jiang^a,b^, Xiaodong Zheng^a,b,^*, Xiaoqi Zeng^a,b^, Lingfeng Kong^b^, Qi Li^b^**

^a^ Institute of Evolution & Marine Biodiversity (IEMB), Ocean University of China, Qingdao 266003, China

^b^ Key Laboratory of Mariculture, Ministry of Education, Ocean University of China, Qingdao 266003, China

*Corresponding author at: Ocean University of China, Qingdao 266003, China

Tel: +86 13708967712, (0532) 82032873

*E-mail address:* xdzheng@ouc.edu.cn

Supplementary Materials

Supplementary Figure S1. Linear comparison of the start and termination codons of *H. chiragra* and *L. lambis* mitochondrial genomes. The numbers following each codon are the frequency of the codon (*H. chiragra* /*L. lambis*).


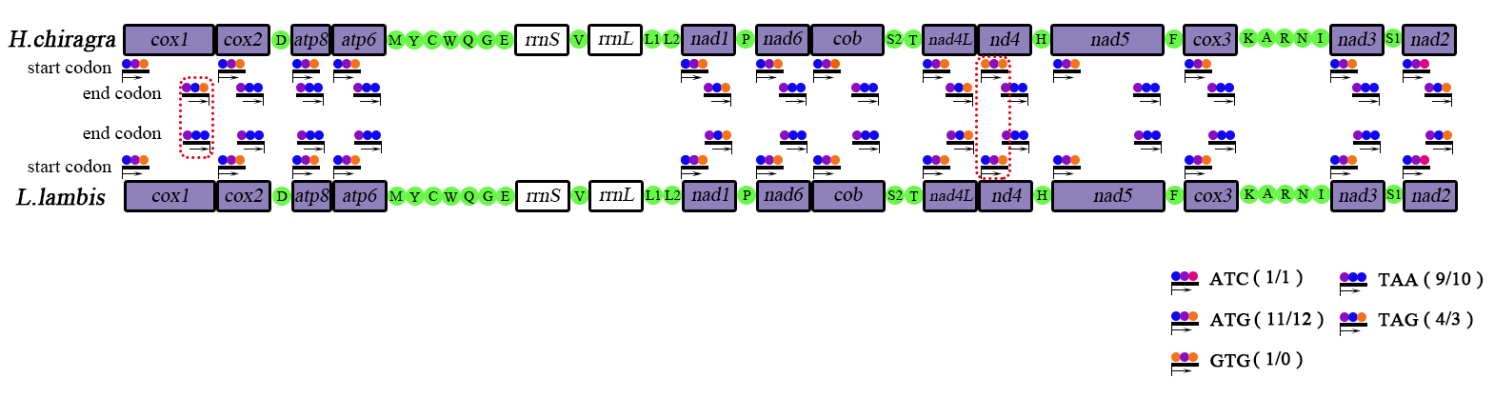


Supplementary Figure S2. Pairwise genetic divergence among the four Strombidae species based on separate and concatenated protein coding genes.


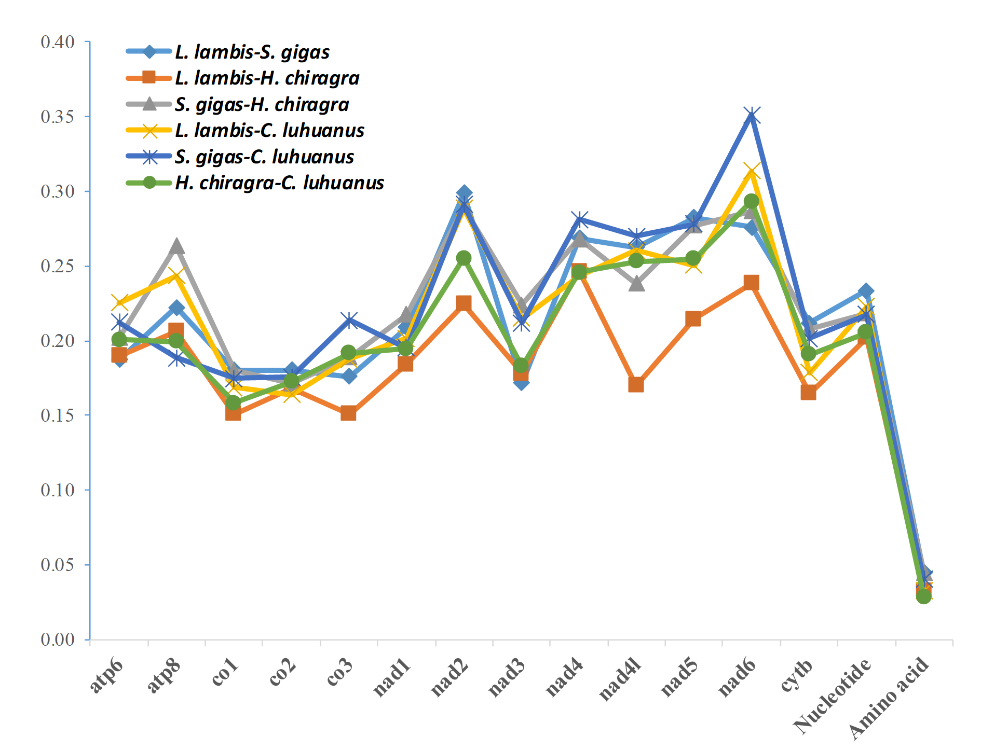


Supplementary Table S1 Primers used in the paper.

| *Harpapo chiragra* |  |
| --- | --- |
| co1-f | 5' GGTCAACAAATCATAAAGATATTGG 3' |
| co1-r | 5' TAAACTTCAGGGTGACCAAAAAATCA 3' |
| co1-atp6-f | 5' CTGCTGTTGAGAGCGGAGTTG 3' |
| co1-atp6-r | 5' TTTGTTTGGTGACCGAGGTT 3' |
| atp6-f | 5' AGTTATAAGTCCACGTTGAGTCT 3' |
| atp6-r | 5' GCAGTATGATAGTTGCCGAT 3' |
| atp6-16s-f | 5' AATTCTTCGTGCCCTAACTCC 3' |
| atp6-16s-r | 5' GTCAAACCATTCATACCAGCC 3' |
| 16s-f | 5' AATCATTGAAAATAATAAACA 3' |
| 16s-r | 5' GAAGATAGAAACTGACCTGGC 3' |
| 16s-cytb-f | 5' TACCGTAGGGATAACAGCATA 3' |
| 16s-cytb-r | 5' ACAGATAAAGAACCAAGAAGC 3' |
| cytb-f | 5' CCGGCACCCTCTAATTTATCTGTAT 3' |
| cytb-r | 5' TGGGATTTTTTCGCCATCTCTAT 3' |
| cytb-co3-f | 5' TATTTATTTCCATATTGGCCG 3' |
| cytb-co3-r | 5' ATTCTCGAATCACATCTCGTC 3' |
| co3-f | 5' GATTAGCTGGTTGATTTCATGGATAT 3' |
| co3-r | 5' GCTCCGTCAGCAATAGTAAAAGAT 3' |
| co3-co1-f | 5' TAGATTGATGGAAGGAGACGG 3' |
| co3-co1-r | 5' AGAAGGAGTAAAGCAGGAGGT 3' |
| *Lambis lambis* |  |
| co1-f | 5' GGTCAACAAATCATAAAGATATTGG 3' |
| co1-r | 5' TAAACTTCAGGGTGACCAAAAAATCA 3' |
| co1-atp6-f | 5' ATACGATGACGAGGGATGCAATT 3' |
| co1-atp6-r | 5' GTGACCGAGGTTTAGGTGGTAGACT 3' |
| atp6-f | 5' AGTTATAAGTCCACGTTGAGTCT 3' |
| atp6-r | 5' GCAGTATGATAGTTGCCGAT 3' |
| atp6-16s-f | 5' GGTCTGCCATTATGACTTTCTT 3' |
| atp6-16s-r | 5' AGCTCGATAGGGTCTTCTTGT 3' |
| 16s-f | 5' AATCATTGAAAATAATAAACA 3' |
| 16s-r | 5' GAAGATAGAAACTGACCTGGC 3' |
| 16s-cytb-f | 5' GCTCATATCGAAAAAAAGGTT 3' |
| 16s-cytb-r | 5' GCAATGGCAAATGGAAGTAAA 3' |
| cytb-f | 5' TGTGCATATTAGTCGTGATGTGAGTT 3' |
| cytb-r | 5' GAATAGAGCGTAAAATGGCATAAGC 3' |
| cytb-co3-f | 5' AACCCGATTTTTCACATTACAT 3' |
| cytb-co3-r | 5' CACCAGCACCATCTCCCTCT 3' |
| co3-f | 5' GATTAGCTGGTTGATTTCATGGATAT 3' |
| co3-r | 5' GCTCCGTCAGCAATAGTAAAAGAT 3' |
| co3-co1-f | 5' CAGATTAATAGAGGGAGATGG 3' |
| co3-co1-r | 5' ATGAAAGAAGTAAAAGCAAGG 3' |

Supplementary Table S2 Taxonomic information and Genbank accession numbers for the species used in this study.

| Superfamily | Species | Accession number |
| --- | --- | --- |
| Stromboidea | *Strombus gigas* | KM245630.1 |
| Tonnoidea | *Conomurex luhuanus* | FJ997214.1 |
|  | *Lambis lambis* | MH115428 (present study) |
|  | *Harpago chiragra* | MH122656 (present study) |
|  | *Galeodea echinophora* | KP716635.1 |
| Littorinoidea | *Littorina saxatilis* | KU952094.1 |
| Naticoidea | *Naticarius hebraeus* | KP716634.1 |
| Rissooidea | *Oncomelania hupensis* | FJ997214.1 |
| Truncatelloidea | *Oncomelania quadrasi* | LC276227.1 |
|  | *Tricula hortensis*, | EU440735.1 |
|  | *Potamopyrgus antipodarum* | GQ996433.1 |
| Vermetoidea | *Potamopyrgus estuarinus* | GQ996415.1 |
|  | *Dendropoma maximum* | HM174253.1 |
| Outgroup | *Eualetes tulipa* | HM174254.1 |
|  | *Thylacodes squamigerus* | HM174255.1 |
|  | *Tegula lividomaculata* | KT207826.1 |
|  | *Tegula brunnea* | JN790613.1 |

Supplementary Table S3 Best partitioned schemes for nucleotide sequences of 13 protein-coding genes for phylogenetic analyses.

| Partition | Alignment length (bp) | DNA substitution models |
| --- | --- | --- |
| *nd5_codon1, nad6_codon1, atp6_codon1, nd4_codon2* | 1153 | GTR+I+G |
| *co3_codon1, cytb_codon2, nd3_codon1, co2_codon2, atp6_codon2* | 1188 | GTR+I+G |
| *co3_codon2, co1_codon3, co2_codon3, cytb_codon3, atp6_codon3* | 1593 | HKY+I+G |
| *atp8_codon2, atp8_codon1, atp8_codon3* | 73 | GTR+G |
| *co1_codon1* | 511 | GTR+I+G |
| *co1_codon2* | 511 | GTR+I+G |
| *co3_codon3, co2_codon1, cytb_codon1, nd3_codon3* | 963 | GTR+I+G |
| *nad6_codon2, nd4l_codon2, nd4l_codon1* | 342 | GTR+I+G |
| *nd1_codon2, nad6_codon3* | 458 | GTR+I+G |
| *nd1_codon1, nd4_codon3* | 608 | GTR+I+G |
| *nd4l_codon3, nd1_codon3* | 405 | GTR+I+G |
| *nd2_codon1, nd2_codon2, nd2_codon3* | 887 | GTR+I+G |
| *nd3_codon2* | 104 | HKY+G |
| *nd5_codon3, nd4_codon1* | 779 | GTR+I+G |
| *nd5_codon2* | 481 | GTR+I+G |
| Concatenation | 10056 |  |

Supplementary Table S4 Best partitioned schemes for amino acid sequences of 13 protein-coding genes for phylogenetic analyses.

| Partition | Alignment length (bp) | DNA substitution models |
| --- | --- | --- |
| *co2, cytb, atp6* | 823 | GTR+I+G |
| *atp8, nd2, nd3* | 338 | HKY+G |
| *co1* | 509 | GTR+I+G |
| *co3, nd1* | 452 | GTR+I+G |
| *nd4, nd4l* | 323 | GTR+I+G |
| *nd5, nd6* | 609 | GTR+I+G |
| Concatenation | 3054 |  |
